# Spiritual Health and Psychological Distress Among Hong Kong Community Lay Leaders

**DOI:** 10.3390/bs14111095

**Published:** 2024-11-14

**Authors:** Shiying Fang, Chi-Hung Leung

**Affiliations:** Department of Special Education & Counselling, The Education University of Hong Kong, Hong Kong, China; fangs@eduhk.hk

**Keywords:** spiritual health, psychological distress, mental health, community lay leaders, COVID-19

## Abstract

Community lay leaders are critical in connecting professional services and general populations in communities. However, limited studies have explored the potential protective factors for psychological health among this group of people. In addition, based on the complex nature of spiritual health, the inconsistent findings of previous studies also suggested that different domains of spiritual health may shape psychological health differently in different contexts and among different socio-demographic groups. Therefore, we assessed the psychological health of Hong Kong community lay leaders after COVID-19 and examined the effects of different domains of spiritual health on psychological distress after controlling for age and gender. Cross-sectional data from 234 Hong Kong community lay leaders aged 18 to 84 were analyzed using structural equation modeling. The results showed that most Hong Kong community lay leaders reported moderate anxiety. In addition, personal and communal (one domain) and transcendental domains of spiritual health were negatively associated with depression, anxiety, and stress, and the environmental domain of spiritual health was positively associated with depression, anxiety, and stress. These findings imply the importance of considering both the positive and negative effects of spiritual health on psychological distress.

## 1. Introduction

The novel coronavirus disease (COVID-19) first occurred in China at the end of 2019 and was considered by the World Health Organization (WHO) a pandemic for the whole world on 11 March 2020 [1]. In Hong Kong, up to 29 January 2023, the Centre for Health Protection of the Development of Health reported 1,223,467 cases tested positive by nucleic acid tests and 1,880,112 cases by RATs for the SARS-CoV-2 virus. Remarkably, 9287 death cases were caused by the coronavirus in 2022 [2]. However, with the spread of COVID-19, the consequent threat was not only to the physical health of those infected but also to the psychological health of general populations around the world.

As an international metropolis, mental disorders are always prevalent in Hong Kong. Early in 2017, the Chinese University of Hong Kong (CUHK) found that 24.3% of full-time working adults in Hong Kong had anxiety and/or depressive symptoms [3]. After the outbreak of COVID-19, surveys conducted by the CUHK from 2019 to 2023 reported that 24.4% of children and adolescents experienced at least one mental health issue over the prior 12 months, and 8.6% of older adults living at home had depression and/or anxiety disorders [4]. Similarly, the Mental Health Association of Hong Kong indicated that the percentage of 2904 Hong Kong residents who scored in the clinical range for depression in 2023 reached 11% and jumped 32% compared with 2020 [5]. According to the advice from the WHO and the assessment results for the local situation, the Hong Kong government announced the lowering of the response level relative to the COVID-19 epidemic from emergency to alert level on 30 May 2023 [6], marking a huge milestone in Hong Kong people’s fight against the pandemic over the prior 3 years. Nonetheless, the fight may not be over yet, and the impacts of the crisis are likely to be deep and persistent.

Identifying the potential protective factors against psychological distress in such a context is important. Previous research has indicated that spiritual health may negatively affect psychological distress. However, the influence of different domains of spiritual health on psychological distress may depend on different contexts [7,8] and socio-demographic groups [9]. To date, when examining the protective role of spiritual health in psychological distress, some studies have focused on groups of adolescents and college students [8,10], while other studies focused on groups of patients [11,12]. With the outbreak of COVID-19, scholars also started to involve healthcare workers in this area [13,14]. To our knowledge, no significant study has focused on community lay leaders and explored the potential protective factors for this group of people’s psychological health. The socio-ecological model underlined the influence of both the level of the individual and the level of the environment per individual development [15]. A perspective based on community offers a unique philosophical orientation for improving health through reaching the public and promoting participation [16]. Similarly, in 2008, the WHO underlined that community-based healthcare helps improve accessibility to public health services, especially in low- and middle-income countries [17]. A systematic review conducted by Mutamba et al. [18] suggested that lay community health workers play a role in establishing a connection between community and professional health services and have the potential to provide psychological interventions. Disadvantaged groups in communities particularly need more attention due to greater exposure and vulnerability to unfavorable social, economic, and environmental circumstances, and community lay leaders play a pivotal and specific role in supporting these groups of people. In previous research, lay leaders usually refer to the leaders who are associated with secular people from religious communities. In this study, we also involved the leaders from general communities in Hong Kong, such as the person in charge of buildings or the chairman of the parents committee and the student body in schools. They are usually voted by the public to serve the public and connect with the officials. They may not have much relevant experience in health practice compared with health professionals. Identifying the protective factors for community lay leaders’ psychological health would be helpful not only for keeping their psychological health up but also for helping them build up a positive mental health environment and maximize community strength. Therefore, in this study, we aimed to assess the psychological health of lay leaders in the Hong Kong community to achieve early identification of psychological distress among this group of people and explore the protective factors against psychological distress after the COVID-19 outbreak.

### 1.1. Hong Kong During COVID-19

The pandemic brought a series of challenges to the people of Hong Kong. The statistics released by the Hong Kong Census and Statistics Department showed that the seasonally adjusted unemployment rate increased from 6.6% in October to December 2020 to 7% in November 2020 to January 2021 [19]. Indeed, to control the spread of the coronavirus, the Hong Kong government imposed a series of measures, such as social distancing and quarantines, which caused an increase in unemployment and an economic recession. Due to the suspension of economic development, some business leaders chose to take a pay cut or reduce their staff; meanwhile, lots of small businesses closed. According to the family stress model [20], job loss as an economic hardship generates economic pressure and negatively influences parents’ psychological condition. Parents’ psychological distress results in dysfunctional parenting practices, and subsequently, child mental health problems increase [21]. In addition, social media exposure may also have been an issue. Extensive coverage by the media of COVID-19 news tended to cause higher mental suffering [22]. During the pandemic, people’s daily lives were usually filled with a large amount of news from both reliable and unreliable sources related to the pandemic, and then the fear of contagion appeared continuously among a certain group of people. Hong Kong families of low socio-economic status were particularly prone to perceived harm during this public health crisis [23].

Apart from the impact of external pressure, the outbreak of COVID-19 was also a challenge for family resilience. Resilience refers to the ability to withstand and rebound from disruptive life challenges and helps individuals respond and recover from dilemmas [24]. During the pandemic, the implementation of home quarantines restricted outdoor entertainment and social activities, making the outbreak of some problems within the family, such as family violence [25]. An American longitudinal study indicated that family chaos increased during COVID-19, and family chaos was associated with an increase in family conflict and a decrease in intimate relationships [26]. Based on family systems theory, the family is an integrated and whole system [27]. Thus, the interdependency of individual family members may cause the transfer of psychological distress and abnormal behaviors within families. Moreover, Bai et al. [28] found that although there was an association between youths’ mental health and parents’ psychological symptoms, parent-child and marital relationships moderated the association. Vulnerable families with a low level of family and community support may have felt a more negative influence from COVID-19-related stressors [29]. To wit, the outbreaks of crises take a higher toll on certain families’ resilience in Hong Kong.

### 1.2. The Role of Spiritual Health During COVID-19

Spiritual health is essential in maintaining and promoting physical and psychological health to achieve happiness and meaning in life even during a pandemic [30]. In the past, health was usually defined by physical, psychological, and social dimensions. Until 1979, the WHO proposed spiritual health as the fourth dimension, along with the previous three dimensions that together comprise health [31]. Bensley [32] suggested that spiritual health’s complex nature makes the construct hard to define. Some scholars have focused on its relationship with a sense of fulfillment in life, while others have underlined values and beliefs regarding community and self. Fisher [33] indicated spiritual health reflects the quality of one’s connection with the self, others, the environment, and God’s power. Further, Sadat Hoseini et al. [34] summarized spiritual health as referring to a set of values of connectedness with the self, others, and the universe guided by a connection with the transcendent and superior. It is a dynamic, conscious, multidimensional, and universal process that becomes activated through spiritual awareness, personal capacity, and potential for transcendence and contributes to spiritual and physical well-being [35].

The maintenance of spiritual health is not localized to certain life stages but is everlasting throughout life; particularly, it influences people’s ability to cope with adversities [36]. Mikaeili and Samadifard [37] suggested that spiritual health may help decrease the level of Iranian teenagers’ suicidal thoughts. Similarly, Anye et al. [38] indicated that the improvement in spiritual health had a beneficial impact on the quality of life among young adults in America. Furthermore, aging is challenging with diverse changes such as physical conditions and social ties. Spirituality could be a resilience strategy for elderly people to cope with health and social problems and achieve well-being [39]. For instance, Lee and Salman [40] and Moxey et al. [41], respectively, found that elderly people’s spiritual health was positively correlated with health-related quality of life in Taiwan, China, and perceptions of social support in Australia. Papathanasiou et al. [42] involved socio-demographic characteristics and reported Greek elderly’s mental disorders were negatively associated with spiritual health, which was influenced by age, place of residence, and health condition. Aydın et al. [43] found that spiritual well-being (one indicator of spiritual health) was not only correlated with fewer mental disorders but also less somatization and more existing meaning in life among the Turkish elderly population. Early in 1999, Mytko and Knight [44] discussed the correlation of spirituality with physical health, mental health, and quality of life. The outbreak of the pandemic in 2019 brought a series of long-term challenges to economic conditions and health among general populations around the world. The persistence of physical problems like fatigue and inactivity and psychological problems like anxiety and depression after infection greatly reduced individuals’ quality of life. Particularly, female patients were relatively more likely to experience frequent mental health symptoms during the pandemic [45]. In fact, the gender difference in the prevalence of mental disorders has been widely discussed by previous research [46,47], and biological, social, and cultural issues may jointly place women in a relatively disadvantaged position compared with men [47]. In addition, a qualitative study conducted in an Iranian military hospital focused on the effect of spiritual health on the treatment of infected patients and indicated that spiritual health helped to change the treatment team and patients’ attitudes towards the disease crisis so that they could consider the crisis and suffering as spiritual experiences [48]. Italian scholar Chirico [49] also suggested during the pandemic that spiritual health could be used to confront mental disorders and economic challenges among general populations and vulnerable groups. In other words, when someone is exposed to stressful situations like health threats, spiritual health can help improve levels of communal and environmental relationships and enable people to cope with crises as integral triggers of human well-being [50].

### 1.3. Spiritual Health and Psychological Distress

There is emerging evidence for the negative association between spiritual health and psychological distress. Gonçalves et al. [51] conducted a systematic review of randomized clinical trials and found that the use of religious/spiritual interventions contributed additional benefits to reducing anxiety symptoms, suggesting the need for religious/spiritual treatment in healthcare. Previous research often focused on exploring the role of spiritual health in healing patients’ physical and psychological distress. For instance, Kandasamy et al. [11] found that advanced cancer patients’ spiritual well-being (an indicator of spiritual health) was negatively correlated with physical and psychological distress and positively correlated with patients’ quality of life. Similarly, Najafi et al. [12] found that spiritual health predicted chronic patients’ psychological disorders, and the improvement in spiritual health was beneficial for reducing the severity of disease symptoms in this group of people. Furthermore, spiritual health may not only directly influence an individual’s psychological health but also serve as an intervening pathway. Kamitsis and Francis [52] suggested spirituality as a significant aspect of people’s experience of nature, which played a mediation role in the association between engagement with nature and psychological well-being. McCormick et al. [53] found that when focusing on young adults with adverse childhood experiences, religious or spiritual struggles served as a mediator to influence this group of people’s mental health symptoms.

With the outbreak of COVID-19, a few studies examined the effect of spiritual health on reducing pandemic-related psychological distress. Lucchetti et al. [54] underlined the significance of religiosity and spirituality and suggested that both of them played an important role in minimizing the mental health consequences of social isolation. Davarinia Motlagh Quchan et al. [14] examined the relationship between COVID-19 anxiety and spiritual health among Iranian nurses and found that nurses with better spiritual health experienced a lower level of COVID-19 anxiety. However, the influence of different domains of spiritual health on psychological distress may depend on the context and socio-demographic factors. Fisher [33] developed the Spiritual Health and Life-Orientation Measure (SHALOM) to assess spiritual health, including personal, communal, environmental, and transcendental domains. Hong Kong scholars Pong et al. [55] combined the personal and communal factors into one domain and proposed a three-factor model, which showed a better fit compared with the original four-factor model. Leung [7] adopted the SHALOM and compared associations between three domains of spiritual health and psychological distress of tertiary students before (2018) and during (2020) the outbreak of COVID-19. The authors found a statistically significant negative relationship between three domains of spiritual health and psychological distress in 2018 and a statistically significant negative relationship between only two domains (communal and personal and environmental) of spiritual health and psychological distress in 2020. Similarly, Leung and Mu [8] compared the influence of spiritual health on the psychological distress of teenagers in China’s Mainland and Hong Kong during COVID-19. They found that both personal and communal and environmental domains negatively affected the psychological distress of Mainland and Hong Kong teenagers, while transcendental domains positively affected psychological distress in Mainland participants. These findings suggested that the impact of COVID-19 may influence the protective role of spiritual health in psychological distress, and the connection between different domains of spiritual health and psychological distress may depend on context. In addition, although the aforementioned studies conducted in Hong Kong had reported a negative association between spiritual health and psychological distress, there were no covariates described. Existing evidence showed a connection with nature that was similar to the environmental domain of spiritual health, which helped reduce psychological distress [56,57,58,59]. However, Dean et al. [9] reported the effect of nature-relatedness on psychological distress, showing different results. They found that nature-relatedness increased stress when controlling for participant characteristics compared with not controlling for participant characteristics, suggesting the significance of accounting for demographic information when exploring the influence of protective factors on people’s psychological health.

### 1.4. The Present Study

Based on the foregoing literature review, this study was designed to examine the effect of spiritual health on psychological distress among Hong Kong community lay leaders in post-COVID-19 after controlling for demographic variables. Age and gender were considered covariates as they had been suggested to affect psychological distress [60,61]. The following research questions were proposed:

1. What is the psychological health of Hong Kong community lay leaders?
2. Do different domains of spiritual health affect psychological distress among Hong Kong community lay leaders after COVID-19 when age and gender are controlled for?

**Figure 1:**
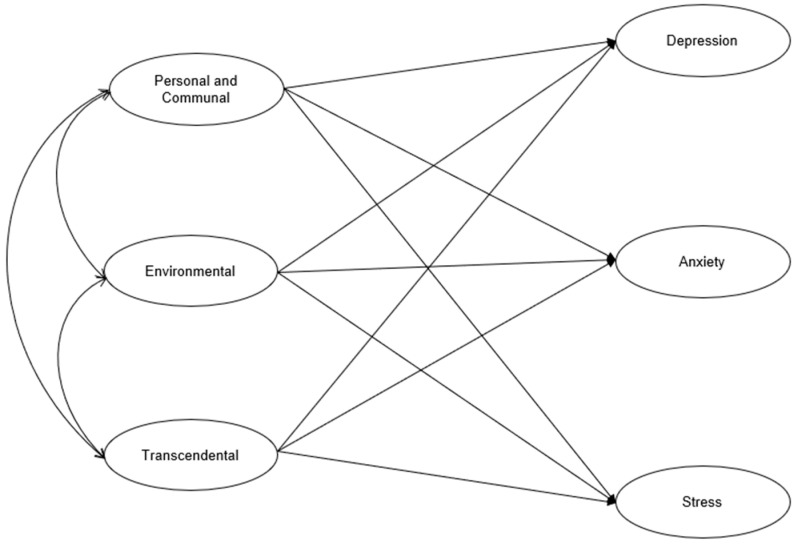
Conceptual model.

As shown in Figure 1, we hypothesized that all the domains of spiritual health would be negatively associated with depression, anxiety, and stress when age and gender were controlled for.

## 2. Method

### 2.1. Participants and Procedures

A total of 234 18–84-year-old community lay leaders in Hong Kong participated in this study. The participants included 66 males (28.21%) and 168 females (71.79%). Most participants (29.49%) had a bachelor’s degree; 87 participants (37.18%) reported having no religious beliefs, and the rest reported Christianity (42.74%), Buddhism (8.97%), Catholicism (6.84%), Taoism (0.85%), and other beliefs (3.42%) affiliations. Demographic information details appear in Table 1.

This study was reviewed and approved by the Human Research Ethics Committee of the Education University of Hong Kong. The participant-signed consent forms were obtained before data collection. Data collection was implemented through the online survey platform Qualtrics. Participants were asked to complete the questionnaire, which included two scales and demographic information. They were told that they had the right to withdraw from the study at any time without any negative consequences.

### 2.2. Measures

#### 2.2.1. The SHALOM

Spiritual health was measured with the SHALOM, including personal, communal, environmental, and transactional domains [33]. This scale consists of 20 items, and the participants were asked to rate the importance and presence of each item in their daily lives on a 5-point Likert scale, ranging from (1) very low to (5) very high. Sample items include “Love for other people”, “Connection with nature”, and “Personal relationship with the Divine”. The Chinese-translated version of the SHALOM was adopted in this study [62]. The SHALOM has demonstrated good reliability and validity in previous studies [8,55]. Based on the findings of Pong et al. [55], in the present study, we combined the personal and communal dimensions into one domain. The three components explained a total of 69.27% of the variance. The Cronbach’s alpha of this scale was 0.95. The details of the results of exploratory factor analysis and reliability analysis of the SHALOM in this study are shown in Table 2.

#### 2.2.2. The DASS-21

Depression, anxiety, and stresswere measured with the Depression, Anxiety, and Stress Scale (DASS-21) [63]. This scale consists of 21 items, and the participants were asked to rate each item on a 4-point Likert scale, ranging from (0) does not apply to me at all to (3) applies to me very much or most of the time to rate the extent to which they experienced each emotion over the last week. Sample items include “I couldn’t seem to experience any positive feeling at all”, “I was aware of dryness of my mouth”, and “I found it hard to wind down”. The Chinese-translated version of the DASS-21 [64] was adopted in the present study. The DASS-21 has demonstrated good reliability and validity in previous studies [7,8]. In the present study, the three components explained a total of 56.90% of the variance. The Cronbach’s alpha of this scale was 0.94. The details of the results of exploratory factor analysis and reliability analysis of the DASS-21 in this study are shown in Table 2.

## 3. Statistical Analysis and Results

All the statistical analysis was performed with SPSS 28.0 and AMOS 28.0. Structural equation modeling (SEM) was used to examine the effects of different domains of spiritual health on depression, anxiety, and stress. Age and gender were controlled for as covariates.

### 3.1. Descriptive Analysis

The status distribution of 234 Hong Kong community lay leaders in psychological distress is shown in Table 3. The percentage of participants who reported extremely severe depression, anxiety, and stress was 2.14%, 7.69%, and 1.71%, respectively. Notably, most participants (36.32%) reported moderate anxiety. The mean, standard deviation, and normality of the study variables are shown in Table 4. According to the normality standard (skewness < 3, kurtosis < 10) proposed by Curran et al. [65], all the variables well met the standard.

### 3.2. Structural Equation Modeling

**Figure 2:**
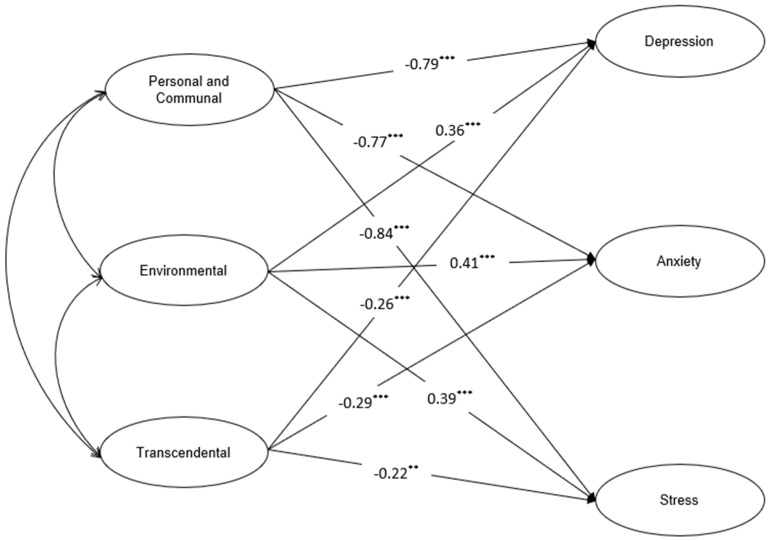
Path of the effects of the personal and communal, environmental, and transactional domains of spiritual health on depression, anxiety, and stress (after controlling for age and gender). *** *p* < 0.001, ** *p* < 0.01.

In SEM, the factor loadings of the measurement model ranged from 0.63 to 0.91 for the SHALOM and from 0.59 to 0.78 for the DASS-21. As presented in Table 5, the structural model also showed a good fit with the data (χ^2^ = 1395.71, df = 842, χ^2^/df = 1.66, CFI = 0.91, RMSEA = 0.05, SRMR = 0.07). As shown in Table 6 and Figure 2, after controlling for the covariates (age and gender), the relationships among the variables in the structural model were all statistically significant. Specifically, the personal and communal domain of spiritual health was negatively associated with depression (β = −0.79, *p* < 0.001), anxiety (β = −0.77, *p* < 0.001), and stress (β = −0.84, *p* < 0.001). The transactional domain of spiritual health was negatively associated with depression (β = −0.26, *p* < 0.001), anxiety (β = −0.29, *p* < 0.001), and stress (β = −0.22, *p* < 0.01). Unexpectedly, the environmental domain of spiritual health was positively associated with depression (β = 0.36, *p* < 0.001), anxiety (β = 0.41, *p* < 0.001), and stress (β = 0.39, *p* < 0.001); 58%, 52%, and 58% of the variance were, respectively, explained in depression, anxiety, and stress (see Figure 2).

## 4. Discussion

### 4.1. Psychological Health of Hong Kong Community Lay Leaders After COVID-19

In total, 2.14%, 7.69%, and 1.71% of Hong Kong community lay leaders in this study reported extremely severe depression, anxiety, and stress, respectively. Also, 36.32% of Hong Kong community lay leaders reported moderate anxiety. One issue that should be noted is that the standard deviations of depression, anxiety, and stress (as shown in Table 4) were somewhat large compared with the three domains (personal and communal, environmental, and transcendental) of SHALOM. This may suggest that the level of psychological health of community lay leaders in Hong Kong was uneven. Leung [7] reported the psychological health status of Hong Kong tertiary students in 2020. Moreover, 14.1%, 25.4%, and 8.8% of participants had extremely severe depression, anxiety, and stress, respectively. Compared with tertiary students, community lay leaders’ general psychological health status was better; however, both of the two populations tended to suffer from anxiety the most. Indeed, after COVID-19, although the government phased out the lockdown policy, COVID-19-related consequences for the economy and society persist. The increase in the unemployment rate and economic recession brought great challenges to people’s lives, especially for disadvantaged groups. In the present study, most participants were female and aged 45 to 64 years, and they were in a critical period of entering old age. A survey conducted by Spytska [66] found that the most common mental disorder of middle-aged women was anxiety disorder. Genetics, financial situation, and unemployment were common causes of mental disorders. In other words, during the outbreak of COVID-19 in Hong Kong, the economic pressure on daily life and threats to physical health were likely to exacerbate typical aging anxiety. In addition, a lack of professional knowledge regarding mental health and the responsibility to connect professional services and communities tended to put the group of Hong Kong community lay leaders in a position of suffering dual pressures of self-healing and supporting others.

### 4.2. Personal and Communal Domains of Spiritual Health and Psychological Distress

Our finding that the personal and communal domains of spiritual health were negatively associated with depression, anxiety, and stress is consistent with most previous studies [7,8,67]. The personal and communal domains measure the extent to which people live in harmony within relationships with themselves and others [33]. In other words, the maintenance of a positive relationship with oneself and others on a spiritual level helps to reduce psychological distress. Michaelson et al. [68] compared the influence of four domains of spiritual health, respectively, on mental health and indicated that “connections to self”, including the sense of meaning in life and experiences of joy in the inner world, could be the most powerful determinant of a positive mental health status. During COVID-19, a longitudinal study by Tutzer et al. [69] also reported the prominent role of meaning in life and peacefulness for people with pre-existing mental health disorders to confront psychological distress. However, Shaver et al. [70] found that young people with stronger connections to themselves faced more distress when they were bullied. The possible explanations were that victimization may challenge some people’s fundamental conceptual systems and disrupt their good intentions when they place particular importance on meanings and happiness in life, and then the conflicts lead to worse mental health outcomes. In addition, Fukui et al. [71] underlined the core role of social networks in the recovery of people with psychiatric disabilities. Similarly, Galloway and Henry [72] reported that social connectedness could serve as a protective factor against depressive symptoms in rural residents. Muse et al. [73] found people who experienced vulnerability in relationships with others were more likely to suffer from anxiety and depression. In the present study, we combined the personal and communal domains into one domain in response to the traditional Confucian values that underscore the transition from personal cultivation to social harmony. Similar to the findings in the aforementioned studies that focused on the role of separate domains, the results in this study showed that the combined domain (personal and communal) of spiritual health also played a strong protective role against psychological distress among Hong Kong community lay leaders.

### 4.3. Transcendental Domain of Spiritual Health and Psychological Distress

Consistent with the findings of Leung and Pong [67], our results showed that the transcendental domain of spiritual health was negatively associated with depression, anxiety, and stress. The transcendental domain measures the extent to which people live in harmony within relationships with something beyond the human level, like the cosmos and God. It involves faith and worship of the mystery of the universe [33]. To wit, the maintenance of a positive relationship with the cosmos or God on a spiritual level may help to reduce psychological distress. For instance, Fukui et al. [71] found people with psychiatric disabilities may benefit from religious attendance. King et al. [74] reported that people who had a spiritual understanding of life but did not follow a religion were more vulnerable to mental disorders. A review conducted by Stewart et al. [75] involved 32 studies and summarized that religion may protect both healthy individuals and patients from suffering anxiety. In the present study, most participants (62.82%) reported having a religious belief system. During COVID-19, the connection to God, including religious beliefs and practices, may work as a source of hope and strength against pandemic-related consequences on mental health. However, Pastwa-Wojciechowska et al. [10] reviewed the role of religion in shaping mental health and underlined both positive and negative aspects of religiosity. Although religiosity may help individuals reduce anxiety and enable them to achieve a sense of self-value, it is also possible to lead to worse mental health due to negative religious beliefs and coping. For instance, people who manifest a religious orientation may experience excessive shame and guilt when they fail to meet religious expectations. Regarding applications of the SHALOM in Hong Kong, Leung and Mu [8] found no statistically significant association between the transcendental domain of spiritual health and psychological distress among tertiary students. Similarly, Leung [7] reported the protective role of the transcendental domain against tertiary students’ psychological distress in 2018, while they found no statistically significant association between the two variables in 2020. Apart from the omission of covariates, the population and study period could be used to account for the inconsistent findings. Although participants’ age was controlled for, the study population in the present study was community lay leaders, and most of them were middle-aged and elderly people. Compared with tertiary students, this group of people is more likely to show stronger subjective religiosity and consistency in religious attendance. A 35-year longitudinal study by Bengtson et al. [76] indicated that the aging of individuals and their progression in life may moderate the association between historical trends and religion and suggested an association between aging and religious intensity. As Sarı [77] explained, aging tends to lead individuals to feel closer to death and consequently be more sensitive to religious activities. Furthermore, due to Hong Kong’s lockdown policy during the pandemic, the restriction of religious practices may have hindered believers from establishing connections with God. After COVID-19, religious practices resumed and worked as a source for people to obtain spiritual power from God and further heal from psychological distress.

### 4.4. Environmental Domain of Spiritual Health and Psychological Distress

An unexpected finding in this study was that the environmental domain of spiritual health was positively associated with depression, anxiety, and stress. The environmental domain measures the extent to which people live in harmony within relationships with the environment [33]. Thus, the maintenance of a positive relationship with the environment on a spiritual level does not necessarily reduce but can increase psychological distress. Existing evidence showed that connection to nature may work as a buffer against cognitive, physical, and mental health disorders. For instance, Dadvand et al. [78] found that lifelong residential surrounding greenness exposure was beneficial for the development of attentional ability. Shuda et al. [79] reviewed seven studies and concluded that there was an inverse relationship between natural exposure and physiologic stress. Regarding the application of the SHALOM in Hong Kong, previous studies also reported a negative association between spiritual connection to the environment and psychological distress among tertiary students; however, no variables were accounted for [7,8,67]. In addition, in 2018, Australian scholars Dean et al. [9] examined the influence of different aspects of nature-relatedness, including self, perspective, and experience, on mental health. The authors found a negative but not statistically significant association between some aspects of nature-relatedness and mental health when the covariates were not accounted for and a statistically significant positive association between nature-relatedness per the self-aspect and depression, anxiety, and stress when the covariates were accounted for. Meanwhile, nature-relatedness and the perspective aspect were also positively associated with stress. Aligning with the findings of Dean et al. [9], the environmental domain of spiritual health measured in the present study underlined that self-identification/connection with nature and view to nature and shared similar concepts and roles with nature-relatedness regarding the self-aspect and perspective-aspect influenced participants’ mental health status. Indeed, the outbreak of COVID-19 not only had huge and long-term impacts on China but also on the whole world. In the early years, it has been indicated that disasters generated in the natural environment could cause emotional anguish such as fear and anxiety for those affected [80,81]. During COVID-19, with the increase of infected individuals, the pandemic-related rumors spread everywhere. The voices such as “The air is poisoned” and “Go outdoors and you will get sick” may cause tremendous panic and reduce people’s willingness to connect with nature. In addition, the subsequent consequences, like deaths and economic recessions, tended to cause a phenomenon of degradation of global living environments. The impact of the pandemic on the environment, including water pollution, increased plastic waste, and chemical contamination, has received attention [82]. In such a context, people with greater environmental concerns may experience increased psychological distress. Shen and Saijo [83] examined the influence of socio-demographic characteristics on individuals’ environmental concerns in Shanghai, China, and indicated that age and educational level were both positively associated with environmental concerns. Compared with young tertiary students who were born during an age of technology, middle-aged community lay leaders were more likely to have different experiences and memories with nature throughout their lives. Furthermore, 21.79%, 29.49%, and 20.94% of the community lay leaders in the present study had associate bachelor’s degrees, bachelor’s degrees, and master’s degrees, respectively. Age/experience and educational background may both lead to this group of people experiencing more environmental concerns and further contribute to increases in psychological distress.

## 5. Conclusions

Community lay leaders’ mental health status is a critical issue for promoting and developing community mental health services. The exploration of the potential protective factors for this group of people’s mental health is vital for not only improving their own mental health but also giving them the strength to support others in their communities. Thus, the present study assessed the psychological health of Hong Kong community lay leaders after COVID-19 and examined the effects of different domains of spiritual health on psychological distress after controlling for age and gender. Taken together, we found that most Hong Kong community lay leaders reported moderate anxiety. In addition, personal and communal, and transcendental domains of spiritual health were negatively associated with depression, anxiety, and stress, and the environmental domain of spiritual health was positively associated with depression, anxiety, and stress.

Our findings highlight the need for a deep understanding of different domains of spiritual health and their associations with mental health among different populations and in different contexts. In addition, the potentially negative aspect of spiritual health is also worthy of serious consideration for further research. The alarmingly high rate of anxiety suggested the significance of paying attention to the psychological health status of Hong Kong community lay leaders. The government should take quick action to solve unemployment issues and achieve economic recovery. Meanwhile, professional mental health training for community lay leaders may help to raise their self-awareness and learn self-regulation for when they suffer from psychological distress. Particularly, it is significant to help vulnerable groups understand the impact of disasters like pandemics, floods, and desertification on the environment correctly and adjust excessive environmental concerns.

Granted, this study had some limitations. First, the nature of this cross-sectional study limited our ability to understand the causality between spiritual health and psychological distress and suggests a need to conduct a longitudinal study to better understand the causal relationships between variables. Second, apart from this quantitative study, a qualitative study could provide relevant mental health practitioners with insights into how spiritual health affects psychological distress specifically. Third, it is imperative to develop intervention strategies to improve mental health based on this study’s findings and examine the interventions’ effectiveness through experiments. Fourth, given the importance of lay leaders in the community and the particularity of their profession, it is necessary to explore the impact of more variables that may correlate with the community lay leaders’ mental health, such as emotion regulation and work experience. Finally, this study used SHALOM to measure spiritual health from three domains. However, when looking back to previous research, the construct of spirituality was always complicated and lacked consistency to a certain extent. Scholars tended to apply different scales to measure and define spiritual health in their studies. Further research should state precisely how the term *spiritual health* was used and then see if the same findings could be duplicated.

## Figures and Tables

**Table 1:** Demographic information (*N* = 234).

		*N*	%
Gender			
	Male	66	28.21
	Female	168	71.79
Age			
	18–24	8	3.42
	25–34	35	14.96
	35–44	36	15.38
	45–54	60	25.64
	55–64	66	28.21
	65–74	28	11.97
	75–84	1	0.43
Education			
	Primary school	6	2.56
	Junior high school	14	5.98
	High school	45	19.23
	Associate bachelor’s	51	21.79
	Bachelor’s	69	29.49
	Master’s or above	49	20.94
Religion			
	Christianity	100	42.74
	Buddhism	21	8.97
	Catholicism	16	6.84
	Taoism	2	0.85
	Other beliefs	8	3.42
	No religious beliefs	87	37.18

**Table 2:** Exploratory factor analysis and reliability of the SHALOM and DASS-21 measures.

		Item	Factor Loading			Total Variance Explained (%)	Cronbach’s Alpha
SHALOM			1	2	3		
	Personal and communal	Q1: Love for other people	0.68				0.94
		Q3: Forgiveness towards others	0.72				
		Q5: Sense of identity	0.77				
		Q8: Trust among individuals	0.78				
		Q9: Self-awareness	0.82				
		Q14: Joy in life	0.67				
		Q16: Inner peace	0.71				
		Q17: Respect for others	0.78				
		Q18: Meaning in life	0.80				
		Q19: Kindness towards other people	0.74				
	Environmental	Q4: Connection with nature		0.80			0.85
		Q7: Appreciation of the breathtaking view		0.49			
		Q10: Oneness with nature		0.76			
		Q12: Harmony with the environment		0.67			
		Q20: Sense of ‘magic’ in the environment		0.75			
	Transactional	Q2: Personal relationship with the Divine			0.81		0.94
		Q6: Worship of the Creator			0.82		
		Q11: Oneness with God			0.89		
		Q13: Peace with God			0.82		
		Q15: Prayer in life			0.84		
	Total					69.27	0.95
DASS-21							
	Depression	Q3: I couldn’t seem to experience any positive feeling at all	0.68				0.87
		Q5: I found it difficult to work up the initiative to do things	0.60				
		Q10: I felt that I had nothing to look forward to	0.73				
		Q13: I felt downhearted and blue	0.57				
		Q16: I was unable to become enthusiastic about anything	0.65				
		Q17: I felt I wasn’t worth much as a person	0.49				
		Q21: I felt that life was meaningless	0.74				
	Anxiety	Q2: I was aware of the dryness of my mouth		0.70			0.86
		Q4: I experienced breathing difficulty (e.g., excessively rapid breath, breathlessness in the absence of physical exertion)		0.61			
		Q7: I experienced trembling (e.g., in the hands)		0.60			
		Q9: I was worried about situations in which I might panic and make a fool of myself		0.64			
		Q15: I felt I was close to panic		0.67			
		Q19: I was aware of the action of my heart in the absence of physical exertion (e.g., sense of heart rate increase, heart missing a beat)		0.70			
		Q20: I felt scared without any good reason		0.60			
	Stress	Q1: I found it hard to wind down			0.64		0.88
		Q6: I tended to over-react to situations			0.65		
		Q8: I felt that I was using a lot of nervous energy			0.65		
		Q11: I found myself getting agitated			0.64		
		Q12: I found it difficult to relax			0.72		
		Q14: I was intolerant of anything that kept me from getting on with what I was doing			0.70		
		Q18: I felt that I was rather touchy			0.69		
	Total					56.90	0.94

**Table 3:** Distribution of the severity of three types of psychological distress (depression, anxiety, and stress).

	Depression	Anxiety	Stress
Normal	0–9	0–7	0–14
	164 (70.09%)	72 (30.77%)	190 (81.20%)
Mild	10–13	8–9	15–18
	25 (10.68%)	41 (17.52%)	18 (7.69%)
Moderate	14–20	10–14	19–25
	37 (15.81%)	85 (36.32%)	16 (6.84%)
Severe	21–27	15–19	26–33
	3 (1.28%)	18 (7.69%)	6 (2.56%)
Extremely severe	28+	20+	34+
	5 (2.14%)	18 (7.69%)	4 (1.71%)

**Table 4:** Mean, standard deviation, and normality of the study variables.

Variables	Mean	Standard Deviation	Skewness	Kurtosis
Personal and communal	3.93	0.64	−0.43	−0.34
Environmental	3.78	0.61	0.11	−0.52
Transactional	3.37	0.98	−0.40	−0.74
Depression	7.09	6.60	1.39	2.08
Anxiety	9.67	6.43	0.78	1.36
Stress	10.39	7.18	0.98	1.30

**Table 5:** Model fit results from confirmatory factor analyses.

	χ^2^	df	χ^2^/df	CFI	RMSEA	SRMR
Acceptable fit threshold				>0.90	<0.08	<0.08
Structural model	1395.71	842	1.66	0.91	0.05	0.07

**Table 6:** The effect of spiritual health on psychological distress (after controlling for age and gender).

	β	S.E.	T	R^2^
Personal and communal → depression	−0.79	0.09	−7.28 ***	
Personal and communal → anxiety	−0.77	0.11	−6.52 ***	
Personal and communal → stress	−0.84	0.10	−7.31 ***	
Environmental → depression	0.36	0.07	3.62 ***	
Environmental → anxiety	0.41	0.08	3.82 ***	
Environmental → stress	0.39	0.07	3.88 ***	
Transactional → depression	−0.26	0.04	−3.42 ***	
Transactional → anxiety	−0.29	0.04	−3.61 ***	
Transactional → stress	−0.22	0.04	−2.91 **	
Depression				0.58
Anxiety				0.52
Stress				0.58

Note: *** *p* < 0.001, ** *p* < 0.01, β = standardized estimate.

## Data Availability

The dataset is available on request from the authors.
